# Fatty Acid Composition and Health Benefits of Some Seed Oils of Emerging Interest

**DOI:** 10.3390/mps8060137

**Published:** 2025-11-08

**Authors:** Teresina Nevigato, Aurora Bocci, Sofia Marica, Roberto Caproni, Maurizio Masci

**Affiliations:** 1 Council for Agricultural Research and Economics (CREA), Research Centre for Food and Nutrition, Via Ardeatina 546, 00178 Rome, Italy; 2 Department of Science, Roma Tre University, Viale G. Marconi 446, 00146 Rome, Italy; aur.bocci1@stud.uniroma3.it (A.B.); sof.marica@stud.uniroma3.it (S.M.)

**Keywords:** fatty acids, food supplements, health benefits, omega-3, pathologies related to the Western diet, gas chromatography, mass spectrometry, AOAC Official Method 991.39

## Abstract

The fatty acid composition of some seed oils from plants of emerging interest was studied. The benefits towards human health were evaluated by taking into account current recommendations regarding dietary intake of essential, polyunsaturated, and monounsaturated fatty acids and by discussing the pathologies for which such fatty acids exert protective action. Species studied were Hemp (*Cannabis sativa*), Flax (*Linum usitatissimum*), Milk Thistle (*Silybum marianum*), Perilla (*Perilla frutescens*), Borage (*Borago officinalis*), and Black Cumin (*Nigella sativa*). Seeds were subjected to cold milling in order to maintain their original nutritional characteristics. Chemical analyses were performed via the dual-detector gas chromatography technique by means of Flame Ionization Detection (FID) and mass spectrometry (MS) and by applying a modified version of the AOAC Official Method 991.39, thanks to which it was possible to obtain the fatty acid composition expressed as mg per gram of oil: such information is not always available in the literature for the species studied here. Comparison with the fatty acid international guidelines about the recommended intakes in g/day was made. This allowed us to evaluate whether such oils are suitable to be used as fatty acid food supplements to rebalance the Western diet, which is shown to be inadequate by numerous studies. Results show that seed oils from *Cannabis sativa*, *Linum usitatissimum*, and *Perilla frutescens* are suitable to be used as food supplements while seed oils from *Silybum marianum*, *Borago officinalis*, and *Nigella sativa* are not. It is important to note that any possible benefits from other parts of the plant (leaves, stems, flowers, and roots) are not studied or questioned by the present research, which focuses solely on fatty acids in the oil extracted from the seeds.

## 1. Introduction

Lipids are one of the pillars of the human diet. Nutritional recommendations from international bodies state that one’s total fat intake must range between 20 and 35% of the Energy Requirement (ER) for it to be adequate for the needs of essential fatty acids and to allow the absorption of lipid soluble vitamins [1]. It is of utmost importance to note that the quality of the fat consumed can either provide invaluable health benefits [1,2,3,4] or cause serious diseases to the human organism [1,5] depending on the fatty acid (FA) composition, since FAs can constitute more than 95% of the total fat by weight [6].

The Western diet currently falls short of following the recommended guidelines about the quality of fat intake in order to maintain good health. This leads to the well-known pathologies of modern life, such as diabetes, cardiovascular diseases, and cancer [4,7].

Human beings evolved consuming a diet with a well-defined composition of fatty acids. This was due to several reasons, such as the availability of certain types of food and intrinsic factors in the primates’ metabolism [8]. Especially in the last hundred years in Western society, there has been a drastic change in diet [4]. On the contrary, the metabolism of the human being has not had time to change so drastically in a hundred years; rather, it has remained the same since it evolved. This state of affairs is likely to lead to dysfunctions and pathologies [8], even serious ones, as they are in fact observed. The most important and biologically significant fatty acids in the human diet are Linoleic (LA or 18:2 ω-6) and α-Linolenic (ALA or 18:3 ω-3). These two FAs are called ‘essential fatty acids’ because the body is not able to synthesize them: they are the precursors of the two main families of polyunsaturated fatty acids (PUFAs), namely, the ω-6 and the ω-3 families which have vital functions in the body [9]. Studies about the dietary intake of PUFAs during the Paleolithic estimate a value of about 0.79 for the ratio Σω-6/Σω-3 [10]. Over the past 100–150 years, there has been an enormous increase in the consumption of ω-6 fatty acids due to the increased intake of vegetable oils from corn, sunflower seeds, safflower seeds, cottonseed, and soybeans [4], together with a diet high in red meat, dairy products, salt, and processed foods [11]. Today, in Western diets, the ratio of Σω-6 to Σω-3 fatty acids ranges from ≈20 to 30:1 or more [4], as Table 1 shows, which is far away from the recommended value that should range from 1:1 to 5:1 [11,12].

Similarly, on the basis of estimates from studies in Paleolithic nutrition and modern-day hunter-gatherer populations, it appears that human beings evolved consuming a diet that was much lower in saturated fatty acids than today’s diet [4].

Given the above and following the massive spread of some non-communicable diseases in the world [13], there is a growing interest in the consumption of food supplements for health purposes. The global dietary supplements market size was valued at USD 192.65 billion in 2024 and is projected to reach USD 414.52 billion by 2033, growing at a Compound Annual Growth Rate (CAGR) of 8.9% from 2025 to 2033 [14]. The European food supplements market was valued at USD 14.95 billion in 2019 and is projected to reach USD 53.53 billion by 2032 [15]. In the far east, Japan has a large functional food market of USD 20 billion per year [16]. Such a turnover means that there is a great variety of products on the market, including those to which synthetic or naturally occurring molecules are added [17,18,19].

**Figure 1:**
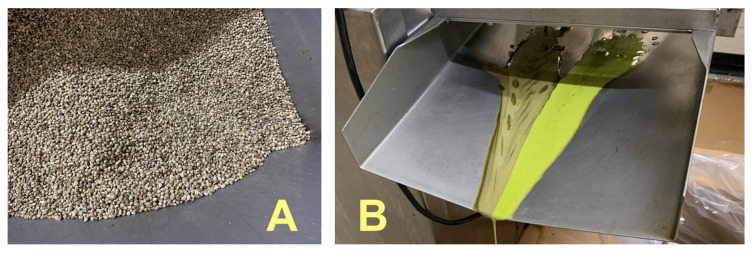
(**A**): Hemp seeds, ‘Codimono’ variety. (**B**): The process of cold milling the seeds shown in (**A**). This oil sample was analyzed in the present work.

In the present research, natural products such as cold-pressed seed oils from increasingly popular plant species are studied (Figure 1). Species investigated were Hemp (*Cannabis sativa*), Flax (*Linum usitatissimum*), Milk Thistle (*Silybum marianum*), Perilla (*Perilla frutescens*), Borage (*Borago officinalis*), and Black Cumin (*Nigella sativa*). They were chosen considering that such oils are widely commercialized as food supplements. The works available in the literature for fatty acids in some of the species mentioned above are few, as is the case of Milk Thistle, for example. Moreover, as a practically constant rule, in almost all studies, only the FA profile is determined, which means the percentage of each fatty acid (FA). To date, no research studies have ascertained absolute FA quantities in all of these species as mg per g of oil and then compared them with the recommended FA quantities as mg/day. Consequently, their potential as food supplements has not been investigated by defining suitable and unsuitable species and indicating minimum daily intake quantities for the suitable ones.

The study carried out in the present work uses gas chromatography–mass spectrometry (GC-MS) to identify all fatty acids present in a sample and takes advantage of the Kinsella procedure [20] to accurately measure the total amount of fatty acids as mg/g present in the same sample. Quantitative measurements performed with gas chromatography–Flame Ionization Detection (GC-FID) complete the analytical method used, which is a modified version of the AOAC Official Method 991.39 [21]. This protocol has already been successfully applied and published [22,23]; nevertheless, method accuracy was rechecked by analyzing a suitable Reference Material provided by the US National Institute of Standards and Technology (NIST).

The main goal of this research was to assess the effectiveness of the oils studied in rebalancing the ω-6/ω-3 ratio by providing a sufficient amount of ω-3 fatty acids, mainly ALA (α-Linolenic acid).

## 2. Materials and Methods

### 2.1. Oil Samples

For the present research, seven different oil samples were selected on the basis of their market diffusion and popularity among consumers in different parts of the world, based on the countries of origin and the main production areas and taking care to include most of the planet. The samples Hemp, of the ‘Futura’ variety, Flax, and Milk Thistle were grown by a trusted small family-run enterprise, which also cold milled the resulting seeds. These samples were grown in the Marche region, Italy. The sample of Hemp (‘Codimono’ variety) was grown in the Veneto region, Italy, by researchers of CREA, Council for Agricultural Research and Economics, Research Centre for Cereal and Industrial Crops (Bologna, Italy). The seeds obtained were cold pressed under our supervision.

Seed oil samples from Perilla, Borage, and Black Cumin were purchased on the market at specialized herbalist shops. When purchasing, we made sure that the label stated the oil was sold as a dietary supplement. The requirements for commercial oils to be analyzed were the cold pressing of the seeds and the proper storage in dark glassware, together with the expiration date: the latter had to be as recent as possible. Additionally, and most importantly, all purchased samples were checked by mass spectrometry for possible degradation peaks, especially of the most degradable fatty acid, ALA. Whenever such peaks appeared in the chromatogram, the purchased sample was not considered for results.

All oil samples were stored in dark glass bottles and, after opening, the bottles were purged with nitrogen and stored at 2 °C to prevent oxidation. As a further measure, TBHQ (tert-Butylhydroquinone, 97%) from Carlo Erba Reagents^®^ (Milan, Italy) was added to the test tubes to prevent sample oxidation during analysis.

### 2.2. Reagents

Methanol, n-hexane, potassium hydroxide, ethanol, hydrochloric acid, TBHQ (tert-Butylhydroquinone, 97%), and chloroform were purchased from Carlo Erba Reagents^®^ (Milan, Italy). Boron trifluoride (BF_3_) methanol solution (14%) was purchased from Merck KGaA^®^ (Darmstadt, Germany). Ethanolic KOH was prepared monthly by dissolving 10 g of potassium hydroxide in 20 mL of distilled water in a refrigerated 100 mL volumetric flask; after the solution became cool, the volume was brought up to the final 100 mL with ethanol.

Individual analytical standards of fatty acids were purchased as methyl esters either from Merck KGaA^®^ (Darmstadt, Germany) or from Larodan^®^ (Solna, Sweden). They were all injected individually to determine their retention time and mass spectrum. The Reference Materials NIST 8183 ‘Botanical oils’ were from National Institute of Standards and Technology (NIST), U.S. Department of Commerce (Gaithersburg, MD, USA). For the purpose of this research, material 8183-3, Flax seed oil, was analyzed.

### 2.3. Derivatization

The glycerides whose oils are constituted were trans-esterified into methyl esters of the individual fatty acids, as already reported [22]. Trans-esterification was conducted by transferring 10 mg of oil in a 20 mL amber tube with a Teflon-lined screw cap. Then 1.5 mL of BF3 14% methanol solution and 1.5 mL of methanol were added. The tube was immersed in a water bath for 18 min at 65 °C and then in a tap water bath, together with the addition of 1.5 mL of distilled water. A total of 3 mL of n-hexane was added only after the solution had cooled. The tube was shaken and the upper hexanic layer was transferred to a 5 mL amber glass vial with a Teflon-lined screw cap and a cone shape at the end, then the solvent was evaporated under a stream of nitrogen. The 3 mL addition of n-hexane in the 20 mL amber tube and the evaporation from the 5 mL vial was repeated another two times. Finally, in the 5 mL vial brought to dryness, 200 µL of n-hexane was introduced: this solution was ready for the gas chromatographic injections.

### 2.4. Instrumental Analysis: GC-MS

Thanks to its great identification power, gas chromatography–mass spectrometry was used for qualitative analyses. By means of GC-MS, all fatty acids present in the oil sample with a signal-to-noise ratio ≥ 3 were searched for. Only after examining the sample via GC-MS was it possible to use the GC-FID technique for quantitative purposes. Note that GC-MS and GC-FID used the same capillary column and the same oven temperature program.

For GC-MS analyses, the instrument used was a Varian 3900 gas chromatograph connected to a Saturn 2100T mass spectrometer equipped with an ion trap analyzer (Varian^®^, Palo Alto, CA, USA). Injections were made in split mode (100:1) with an injection volume of 0.4 µL and by using 1 µL of n-hexane as a plug. The capillary column installed was a CP-WAX 52 CB (60 m × 0.32 mm I.D., 0.50 µm film thickness) from Chrompack^®^, Middelburg, the Netherlands. The injector temperature was 220 °C. The column oven temperature was programmed as follows: initial temperature 50 °C, 2 min hold; rising to 140 °C at a rate of 22.5 °C/min, 0 min hold; and rising to 228 °C at a rate of 2 °C/min, 44 min hold. Helium flow, as the carrier gas, was programmed from 1 mL/min (initial) to 2.5 mL/min (final). Transfer line and ion trap temperatures were 220 and 180 °C, respectively. Full scan mass spectra were achieved through EI (electron ionization) mode at 70 eV, with an emission current of 10 µA in the acquisition range 40–440 *m*/*z*.

**Figure 2:**
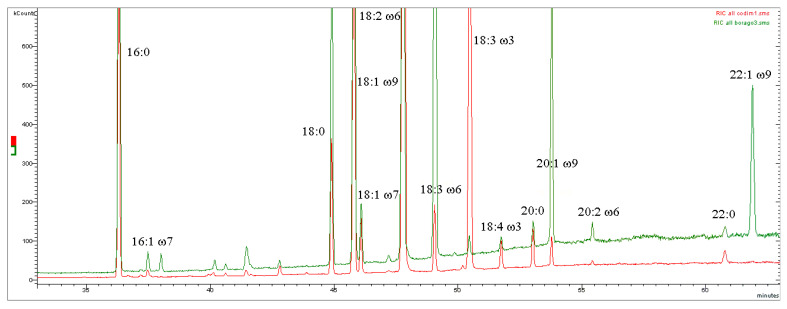
Gas chromatograms obtained by mass spectrometry (GC-MS) in the time range 33–63 min. Overlapping between the sample Hemp seed oil of the ‘Codimono’ variety (analysis number 1) traced in red and the sample Borage seed oil (analysis number 3) traced in green. Fatty acid methyl esters. The high concentration of ALA in Hemp seed oil makes it suitable for rebalancing the LA/ALA ratio in the diet, unlike Borage seed oil (see Section 4.3).

**Figure 3:**
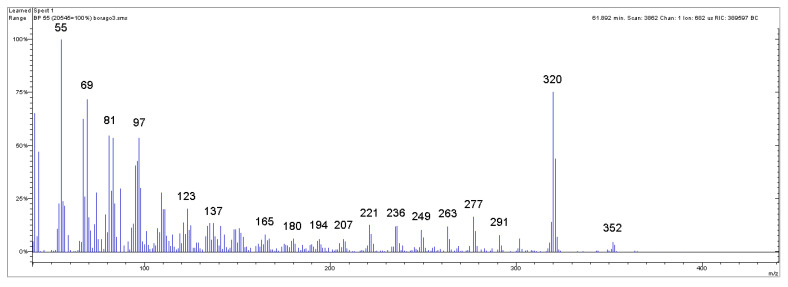
Mass spectrum of the peak related to 22:1 ω9 (erucic acid methyl ester) in Borage seed oil of Figure 2. The characteristic intense ionic fragment at 320 *m*/*z* is clearly visible, followed by the fragment at 352 *m*/*z*, which represents the molecular ion of the erucic acid methyl ester.

Examples of mass chromatograms and mass spectra are reported in Figure 2 and Figure 3 and in the Supplementary Materials (Figures S1–S5).

### 2.5. Instrumental Analysis: GC-FID

The instrument used was a 6890 Agilent^®^ gas chromatograph (Agilent Technologies, Santa Clara, CA, USA) equipped with a flame ionization detector (GC-FID). The column oven temperature program, the capillary column installed, and the Helium flow program were the same as GC-MS. Injections were made in split mode (40:1) with an injection volume of 1 µL. The injector temperature was 220 °C and the detector temperature was 275 °C.

**Figure 4:**
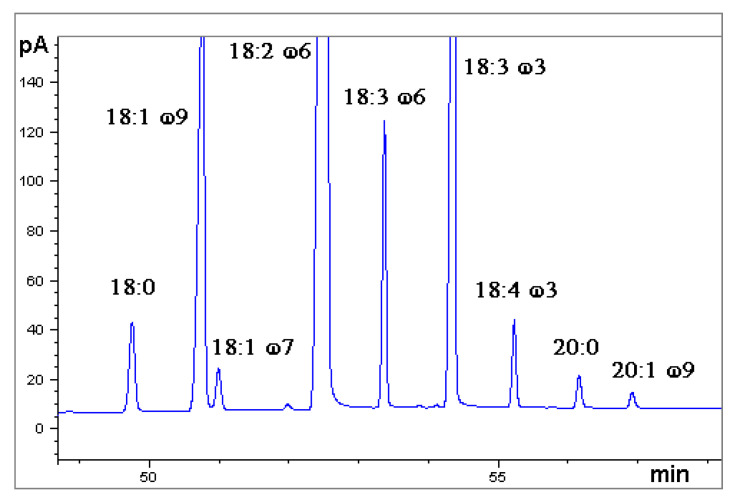
Gas chromatogram obtained by Flame Ionization Detection (GC-FID). Sample: Hemp seed oil, ‘Futura’ variety (analysis number 1), in the time range 49–58 min. Fatty acid methyl esters.

A chromatogram obtained by GC-FID is shown in Figure 4.

### 2.6. The Kinsella Procedure

The Kinsella procedure was used to measure the total content of fatty acids as mg per g of oil to be related with the percentages determined by GC-FID, as already reported [22,23]. Briefly said, an aliquot of about 100 mg of oil were subjected to saponification using 10% alcoholic KOH. The non-saponifiable material was extracted with n-hexane and discarded. The residual soaps were acidified to pH 1.5 in order to reconstitute free fatty acids. The free fatty acids were extracted with n-hexane and dried in a tared vial. The weight of total fatty acids was determined by difference.

### 2.7. Summary of the Method

The protocol described above, from Section 2.3, Section 2.4, Section 2.5 and Section 2.6, was always performed in triplicate. An average of 25 fatty acids were analyzed in each sample for a total of about 75 qualitative determinations by GC-MS and 75 quantitative determinations by GC-FID for each seed oil. The concentrations of the individual fatty acids can be found in the Supplementary Materials, Tables S1–S14.

The content of each fatty acid as mg per g of oil was obtained directly by multiplying the percentage observed via GC-FID by the total fatty acid content measured with the Kinsella procedure without the need for any conversion factor.

The observed Limit of Quantitation of the method was 0.01 mg/g.

## 3. Results

### 3.1. Accuracy Verification

To ensure the accuracy of the method, a Reference Material was analyzed (NIST RM 8183-3, Flax seed oil). The Reference Material Information Sheet reports the results as the mean ± 2σ, and the same was performed here for all the results of the present research (Supplementary Materials, Tables S1–S14). The symbol 2σ stands for twice the standard deviation.

From the statistical point of view, the interval ‘mean ± 2σ’ indicates data dispersion among replicates. The result for a certain fatty acid obtained in the present work is considered to be in agreement with the value declared by NIST when the two intervals of data dispersion intersect. This occurs in 12 out of 14 cases, as shown in Table 2. Only for the two minor fatty acids 12:0 and 17:0, at trace level, the intervals do not intersect.

From the above, therefore, we can say that results agreed with RM/NIST within the observed dispersion (±2σ; n = 3), indicating good analytical reproducibility.

### 3.2. Results Obtained from the Oil Samples Analyzed

Fatty acid compositions of the oil samples analyzed are summarized in Table 3 (percentages) and in Table 4 (mg per gram of oil). Table 3 and Table 4 report the most important parameters related to human health: Linoleic acid (LA), α-Linolenic acid (ALA), LA/ALA ratio, Σω6, Σω3, Σω6/Σω3 ratio, ΣPUFA (polyunsaturated fatty acids), ΣMUFA (monounsaturated fatty acids), and ΣSFA (saturated fatty acids). Detailed results for each detected fatty acid in the oils studied are reported in Tables S1–S14 of the Supplementary Materials.

## 4. Discussion

### 4.1. Recommended Daily Intake of Fatty Acids by International Guidelines

Dysfunctions and pathologies related to incorrect intake of fatty acids are numerous and can also be serious. On the other hand, it has been demonstrated that the prevention of these same pathologies and the maintenance of a state of good health can be effectively obtained with the correct intake of fatty acids through diet.

A diet rich in saturated fatty acids has negative effects on health: higher dietary intakes of major SFAs are associated with an increased risk of coronary heart disease [5] and are associated with all-cause mortality, heart disease mortality, and cerebrovascular disease mortality [24]. In addition, saturated fatty acids tend to raise total blood cholesterol levels even more than dietary cholesterol intake itself [25]. On the contrary, a diet rich in unsaturated fatty acids has universally recognized beneficial effects on health [26]. Significant amounts of saturated fatty acids are found in meat, lard, butter, and margarine, while relevant amounts of unsaturated fats can be obtained from vegetable oils, nuts, seeds, and fish.

Among unsaturated fatty acids, the essential ones play fundamental metabolic roles. They are called ‘essential’ because the human organism cannot synthesize them. There are two essential fatty acids and both are PUFAs: Linoleic acid (LA or 18:2 ω-6) and α-Linolenic acid (ALA or 18:3 ω-3). Through specific metabolic pathways, they are converted into other longer chain PUFAs.

**Figure 5:**
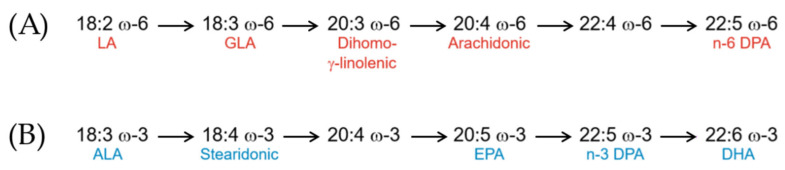
(**A**) Metabolic pathway of ω-6 fatty acids and (**B**) metabolic pathway of ω-3 fatty acids [9].

Figure 5 shows how LA and ALA are the precursors of the two main families of PUFAs, namely the ω-6 and the ω-3 families. LA and ALA are metabolized by the same microsomal enzyme system by alternating desaturation and elongation to make two cascades of metabolic products up to 22 or more carbons long. The two pathways compete for the same enzyme sites (elongase and desaturase) so that the excess activity of one pathway inhibits the activity of the other. Therefore, as the intake of LA increases, metabolic products of ALA are suppressed and vice versa [9].

Both families give rise to important molecules for the organism. From Linoleic acid comes Arachidonic and Dihomo-γ-linolenic acids, which represent the substrate for the production of eicosanoids in tissues. Linoleic acid is a component of membrane structural lipids and is also important in cell-signaling pathways. α-Linolenic acid is the precursor of Eicosapentaenoic acid (EPA or 20:5 ω-3), DHA (Docosahexaenoic acid or 22:6 ω-3), and n-3 eicosanoids, which have been shown to have beneficial effects in preventing coronary heart disease, arrythmias, and/or thrombosis [27]. EPA and DHA are also known for their beneficial effects with regard to cognitive health [28]. It is therefore of primary importance that the intake of omega-3 and omega-6 is balanced to prevent unwanted effects. It should be noted that mammalian cells cannot convert omega-6 to omega-3 fatty acids because they lack the converting enzyme, omega-3 desaturase [12]. Therefore, omega-6 and omega-3 fatty acids are not interconvertible, are metabolically and functionally distinct, and often have important opposing physiological effects, which is why their balance in one’s diet is important [12].

International guidelines recommend an intake of omega-6 no more than five times that of omega-3 [11,12], but the current Western diet far exceeds this recommendation, with omega-6 intakes reaching up to 50 times that of omega-3 (Table 1). This causes widespread pathologies because an excess of omega-6 fatty acids in the diet increases the risk of obesity and metabolic syndrome [12,29], increases insulin resistance [7] and causes EPA and DHA deficiencies [28]. Moreover, a highly unbalanced omega-6/omega-3 ratio in favor of omega-6 PUFAs has been observed to be pro-thrombotic and pro-inflammatory [12].

From the above-mentioned health considerations, the recommendations for intake by international organizations are derived. Recommendations take into account the individual daily Energy Requirement (ER) based on sex, age, Physical Activity Level (PAL), Basal Metabolic Rate (BMR), and Body Mass Index (BMI). BMI is calculated as weight (in kilograms) divided by height squared (in meters). The acceptable BMI range for adults is 18.5 to 24.9 [27,30]. Once the Energy Requirement in kcal/day has been established, the recommended amounts of each fatty acid are expressed in %E. Considering that 1 g of fat provides 9 kcal, it is quite easy to calculate the recommended g/day of each fatty acid.

Table 5 and Table 6 show the optimal intake quantities for two example subjects: a man, 35 years old, 1.79 m tall, 80 kg weight, with a sedentary lifestyle and a woman, 25 years old, 1.64 m tall, 50 kg weight, with an active lifestyle. Complete tables for subjects in a wide range of age, PAL, BMR, and BMI are available from the Food and Agriculture Organization of the United Nations [1,30] or from the Food and Nutrition Board, Institute of Medicine National Academies, Washington, DC, United States [27].

It is possible to observe in Table 5 and Table 6 how the ratio LA/ALA is within the limit of 5, as generally recommended for the ratio between the omega-6 precursor (LA) and the omega-3 precursor (ALA) [4]. In fact, for the subject ‘man, 35 years old’, the minimum recommended LA intake is 7 g/day, and the minimum recommended ALA intake is 1.4 g/day with a resulting ratio of 7/1.4 = 5. The same is valid for the subject ‘woman, 25 years old’ for which the ratio is 6/1.2 = 5.

### 4.2. Suitability of the Oils Studied as Fatty Acid Food Supplements

By comparing the results of the analyses carried out in the present research with the recommended fatty acid intakes, it is possible to judge the quality of the oils examined and evaluate their suitability as fatty acid food supplements.

**Figure 6:**
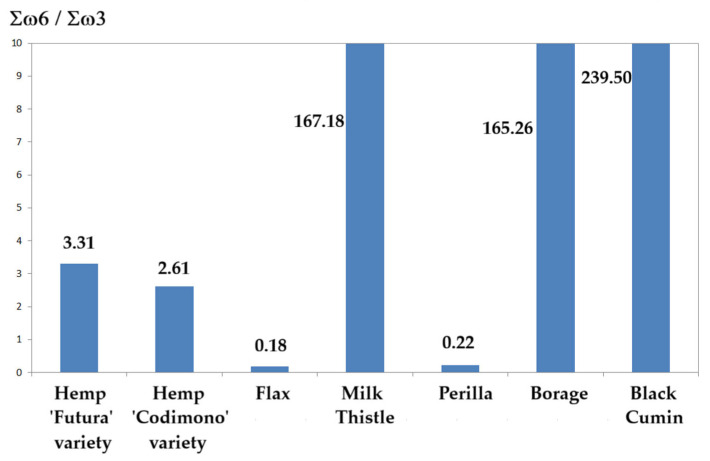
Omega-6 to omega-3 ratio measured in the oil samples analyzed.

Figure 6 shows the values of Σω6/Σω3 measured in the seed oils studied (Table 3 and Table 4). It is evident how the values observed for Milk Thistle, Borage, and Black Cumin (167.18, 165.26, and 239.50) are not helpful in rebalancing the diet. Indeed, the recommended Σω6/Σω3 value in the total daily diet should range between 1 and 5 [4,11,12]. This makes these oils unsuitable as fatty acid food supplements in Western countries, where the diet has precisely this defect, that is, an omega-6 to omega-3 ratio that is too high. The Σω6/Σω3 values in Hemp, Flax, and Perilla are, instead, very helpful in rebalancing the diet. Such a situation is caused by the low omega-3 content in Milk Thistle, Borage, and Black Cumin (0.29, 0.37, and 0.26%) versus the content in Hemp (17.87 and 21.82%), in Flax (63.60%), and in Perilla (62.66%), as shown in Table 3. From this point of view, Hemp seed oil, Flax seed oil, and Perilla seed oil have a high nutritional value if we consider that other commonly used oils such as corn oil, sunflower oil, and peanut oil have omega-3 contents below 1%, below 0.5%, and traces, respectively. It should be noted in Table 3 and Table 4 that the LA/ALA ratio has about the same value as the Σω6/Σω3 ratio in each sample analyzed here: this is why the fatty acid LA in these oils largely predominates among the omega-6s just as ALA largely predominates among the omega-3s.

In regard to the content of unsaturated FAs, all oils analyzed showed good quantities. However, Hemp, Flax, and Perilla exhibited more favorable values of polyunsaturated fatty acids, confirming their high nutritional value. The value for ΣPUFA is 77.00, 78.76, 75.20, and 76.33% for Hemp, Flax, and Perilla (Table 3), while for Milk Thistle, Borage, and Black Cumin, the ΣPUFA is 48.50, 61.91, and 62.32%, respectively.

### 4.3. Covering FA Needs with Hemp, Flax, and Perilla Seed Oils

The European Directive 2002/46/EC says that dietary supplements are foodstuffs where the purpose of which is to supplement the normal diet and which are designed to be taken in measured, small-unit quantities [31].

As a small-unit quantity, we consider a teaspoon whose content is about 4 g of oil. As seen before, Table 5 and Table 6 report the recommended fatty acid daily intakes for two example subjects. Optimal intake quantities of the studied oils can be obtained by comparing the recommended values in Table 5 and Table 6 with the measured concentrations in Table 3 and Table 4. For example, the Flax seed oil in Table 4 contains 0.59 g of ALA per g of oil, that means 2.38 g of ALA in a teaspoon of 4 g and 1.19 g of ALA in half a teaspoon. We observe that the recommended ALA minimum daily intake in Table 6 for the subject woman, 25 years old, 1.64 m tall, 50 kg weight, and with an active lifestyle is 1.2 g. From this, we deduce that half a teaspoon of Flax seed oil alone provides 99% of the daily requirement of ALA for the subject. From the same half teaspoon of Flax seed oil comes 0.17 g of saturated FAs (Table 4), which represent only 0.81% of the 21 g daily that should not be exceeded by the subject (Table 5). Such calculations demonstrate that Flax seed oil is suitable as a food supplement. Perilla seed oil shows very similar amounts of fatty acids to Flax, with calculations and conclusions very similar to those made for Flax seed oil.

Considering that the teaspoon of oil is not the only food consumed and that other fatty acids certainly come from other foods in the daily diet, Hemp seed oil can also be considered as a good dietary supplement. For example, Hemp seed oil of the ‘Codimono’ variety contains 0.196 g of ALA per g of oil (Table 4), which means 0.78 g of ALA in a teaspoon of 4 g. Therefore, a teaspoon of Hemp seed oil of the ‘Codimono’ variety provides 65% of the daily requirement of ALA (1.2 g) for the subject ‘woman, 25 years old, 1.64 m tall, 50 kg weight, with an active lifestyle’.

For this study, we consider an oil adequate if a 1-teaspoon serving (≈4 g) covers at least 40–50% of the daily ALA target when used to replace a commonly used low-ALA fat (e.g., refined sunflower oil) without increasing the total caloric intake.

In Table 7 the above-exposed calculations are shown for ALA, for the two example subjects, and for the three oils suitable as food supplements.

Daily coverage of ALA needs, as exposed in Table 7, are valid under ideal storage conditions for the oil. Indeed, ALA is one of the most degradable fatty acids, depending on the temperature, light, and the time elapsed since production, even in the case of commercial samples to which some antioxidants are generally added. Therefore, in everyday use, coverage estimates per serving from Table 7 should be interpreted as ranges and may vary with oil freshness and storage conditions.

## 5. Conclusions

In the present research, the fatty acid composition of some seed oils coming from plants of growing interest such as Hemp, Flax, Milk Thistle, Perilla, Borage, and Black Cumin was analyzed. These oils are widely present on the market and are sold as food supplements. To judge their suitability as fatty acid food supplements, an accurate qualitative and quantitative analysis was conducted by using a double gas chromatography technique with mass spectrometry and Flame Ionization Detection. The data obtained were then compared with the nutritional recommendations from the international organizations responsible for health protection.

The final outcome was that some oils marketed as fatty acid food supplements are unsuitable for this function. They are Milk Thistle seed oil, Borage seed oil, and Black Cumin seed oil.

On the contrary, Hemp, Flax, and Perilla seed oils are suitable as fatty acid food supplements. The present work demonstrates that small daily amounts of these oils are sufficient to cover the requirement of the essential fatty acid α-Linolenic, which is an omega-3 fatty acid and the precursor of all omega-3 fatty acids; these are quite deficient in the Western diet. The only other available food source of omega-3 is represented by fish products, which, however, not everyone can consume adequately or want to consume (see, for example, vegetarians). Therefore, the availability of vegetable oils capable of providing an adequate quantity of omega-3s, even with small daily intakes, should be considered as an important resource. It should be emphasized that the high content of omega-3s (20–60%) in the three species Hemp, Flax, and Perilla is a fairly rare characteristic in vegetable oils, which are generally poor in them, such as corn oil, sunflower oil, and peanut oil.

## Figures and Tables

**Table 1:** The Σω-6/Σω-3 ratios in the diet of different populations (excerpt from Simopoulos, 2016 [12]).

Population	Σω-6/Σω-3
Paleolithic	0.79
Greece prior to 1960	1.00–2.00
Current Japan	4.00
Current India, rural	5–6.1
Current UK and northern Europe	15.00
Current US	16.74
Current India, urban	38–50

**Table 2:** Analysis of the NIST Reference Material 8183-3.

	NIST Reference Material: Declared Values (mg/g)		Measured Values in the Present Research ^a^ (mg/g)		
	Mean ± 2σ	Interval of the Data Dispersion	Mean ± 2σ	Interval of the Data Dispersion	Intersection of the Data Dispersion Intervals ^b^
12:0	0.016 ± 0.001	0.015–0.017	0.04 ± 0.01	0.03–0.05	No
14:0	0.271 ± 0.008	0.263–0.279	0.27 ± 0.04	0.23–0.31	Yes
15:0	0.151 ± 0.016	0.135–0.167	0.15 ± 0.02	0.13–0.17	Yes
16:0	44.8 ± 5.0	39.8–49.8	42.21 ± 3.46	38.75–45.67	Yes
16:1 ω-7	0.383 ± 0.031	0.352–0.414	0.39 ± 0.03	0.36–0.42	Yes
17:0	0.212 ± 0.011	0.201–0.223	0.43 ± 0.08	0.35–0.51	No
18:0	30.4 ± 2.4	28.0–32.8	36.40 ± 4.46	31.94–40.86	Yes
18:1 ω-9	165.7 ± 6.2	159.5–171.9	174.05 ± 14.20	159.85–188.25	Yes
18:1 ω-7	5.61 ± 0.16	5.45–5.77	6.03 ± 1.01	5.02–7.04	Yes
18:2 ω-6	171 ± 11	160–182	175.70 ± 0.53	175.17–176.23	Yes
18:3 ω-3	579 ± 30	549–609	544.27 ± 12.40	531.87–556.67	Yes
20:0	1.04 ± 0.15	0.89–1.19	0.78 ± 0.14	0.64–0.92	Yes
22:0	0.62 ± 0.13	0.49–0.75	0.52 ± 0.18	0.34–0.70	Yes
24:0	0.308 ± 0.059	0.249–0.367	0.34 ± 0.04	0.30–0.38	Yes

^a^ n = 3 independent measurements. ^b^ ‘No’ = the intervals do not intersect. ‘Yes’ = the intervals intersect. σ = Standard deviation.

**Table 3:** Overview of fatty acid composition in the oils analyzed (percentages ^a^). Mean values. Details are reported in Supplementary Materials (Tables S1–S14).

Sample	LA	ALA	LA/ALA	Σω6	Σω3	Σω6/Σω3	ΣPUFA	ΣMUFA	ΣSFA
Hemp seed oil ^b^	54.64	16.47	3.32	59.13	17.87	3.31	77.00	12.71	10.05
Hemp seed oil ^c^	55.30	21.19	2.61	56.94	21.82	2.61	78.76	11.69	9.20
Flax seed oil	11.55	63.56	0.18	11.60	63.60	0.18	75.20	15.46	8.98
Milk Thistle seed oil	48.17	0.29	167.06	48.21	0.29	167.18	48.50	34.00	17.21
Perilla seed oil	13.56	62.63	0.22	13.66	62.66	0.22	76.33	15.34	7.77
Borage seed oil	38.41	0.23	164.76	61.54	0.37	165.26	61.91	23.53	14.32
Black Cumin seed oil	59.28	0.26	228.78	62.06	0.26	239.50	62.32	23.04	14.37

^a^ LA/ALA and Σω6/Σω3 ratios are dimensionless. ^b^ ‘Futura’ variety. ^c^ ‘Codimono’ variety.

**Table 4:** Overview of fatty acid composition in the oils analyzed (mg per g of oil ^a^). Mean values. Details are reported in Supplementary Materials (Tables S1–S14).

Sample	LA	ALA	LA/ALA	Σω6	Σω3	Σω6/Σω3	ΣPUFA	ΣMUFA	ΣSFA
Hemp seed oil ^b^	514.90	155.18	3.32	557.20	168.43	3.31	725.63	119.78	94.72
Hemp seed oil ^c^	511.00	195.78	2.61	526.13	201.65	2.61	727.78	107.97	85.02
Flax seed oil	108.00	594.31	0.18	108.43	594.68	0.18	703.11	144.50	83.99
Milk Thistle seed oil	458.14	2.74	167.06	458.45	2.74	167.18	461.19	323.37	163.69
Perilla seed oil	126.88	586.20	0.22	127.90	586.50	0.22	714.40	143.62	72.69
Borage seed oil	361.78	2.20	164.76	579.73	3.51	165.26	583.24	221.61	134.86
Black Cumin seed oil	532.93	2.33	228.78	557.89	2.33	239.50	560.22	207.13	129.16

^a^ LA/ALA and Σω6/Σω3 ratios are dimensionless. ^b^ ‘Futura’ variety. ^c^ ‘Codimono’ variety.

**Table 5:** Recommended intakes (g/day) of total fat and saturated fatty acids (SFAs) by international guidelines [1,30].

	Minimum Fat Intake		Maximum Fat Intake		SFA Intake	
Subject	Man *	Woman **	Man *	Woman **	Man *	Woman **
Energy requirement (kcal/day)	2600	2150	2600	2150	2600	2150
Recommended value	15%E	15%E	30–35%E	30–35%E	<10%E	<10%E
Energy from the recommended value (kcal/day)	390	322.5	780–910	645–752	<260	<215
Resulting recommended intake (g/day)	43	36	87–101	72–84	<29	<21

* Man, 35 years old, 1.79 m tall, 80 kg weight, and sedentary lifestyle. ** Woman, 25 years old, 1.64 m tall, 50 kg weight, and active lifestyle.

**Table 6:** Recommended intakes (g/day) of Linoleic acid (LA), α-Linolenic acid (ALA), and polyunsaturated fatty acids (PUFAs) by international guidelines [1,30].

	Minimum LA Intake		Minimum ALA Intake		PUFA Intake	
Subject	Man *	Woman **	Man *	Woman **	Man *	Woman **
Energy requirement (kcal/day)	2600	2150	2600	2150	2600	2150
Recommended value	2.5%E	2.5%E	0.5%E	0.5%E	6–11%E	6–11%E
Energy from the recommended value (kcal/day)	65	54	13	11	156–286	129–236
Resulting recommended intake (g/day)	7	6	1.4	1.2	17–32	14–26

* Man, 35 years old, 1.79 m tall, 80 kg weight, and sedentary lifestyle. ** Woman, 25 years old, 1.64 m tall, 50 kg weight, and active lifestyle.

**Table 7:** Daily coverage of ALA needs with one teaspoon of Hemp, Flax, and Perilla seed oil.

	Recommended ALA Daily Intake (g/Day)	ALA Content in 1 Teaspoon (g)				Daily Coverage of ALA Need with 1 Teaspoon			
		Hemp ^a^	Hemp ^b^	Flax	Perilla	Hemp ^a^	Hemp ^b^	Flax	Perilla
Man *	1.4	0.62	0.78	2.38	2.34	44%	56%	170%	167%
Woman **	1.2	0.62	0.78	2.38	2.34	52%	65%	198%	195%

* Man, 35 years old, 1.79 m tall, 80 kg weight, and sedentary lifestyle. ** Woman, 25 years old, 1.64 m tall, 50 kg weight, and active lifestyle. ^a^ ‘Futura’ variety. ^b^ ‘Codimono’ variety.

## Data Availability

Data is contained within the article or Supplementary Material.

## Supplementary Materials

The following supporting information can be downloaded at https://www.mdpi.com/article/10.3390/mps8060137/s1, Tables S1–S14: detailed fatty acid composition of Hemp (*Cannabis sativa*), Flax (*Linum usitatissimum*), Milk Thistle (*Silybum marianum*), Perilla (*Perilla frutescens*), Borage (*Borago officinalis*), and Black Cumin (*Nigella sativa*) seed oils; Figures S1–S5: Gas chromatograms and mass spectra.
