# Supplementary material for: Fatty Acid Composition and Health Benefits of Some Seed Oils of Emerging Interest
Source: Methods Protoc. 2025 Nov 8;8(6):137. doi: 10.3390/mps8060137 (PMC12641930; doi:10.3390/mps8060137)
Supplement: Supplementary file 1 [file mps-08-00137-s001.zip › mps-3904325-supplementary.pdf]

Fatty acid composition and health benefits of some seed oils of emerging interest

Teresina Nevigato*[1], Aurora Bocci[2], Sofia Marica[2], Roberto Caproni[1], Maurizio Masci*[1]

*Corresponding authors: teresina.nevigato@crea.gov.it
maurizio.masci@crea.gov.it

[1]CREA, Council for agricultural research and economics.
Research Centre for Food and Nutrition, via Ardeatina 546 - 00178 Rome (Italy)

[2]Roma Tre University, Department of Science, viale G. Marconi 446 - 00146 Rome (Italy)

Summary. Edible oils from the seeds of six different plant species were analyzed for their fatty acid composition. Such products are sold as food supplements. Species investigated were Hemp (*Cannabis sativa*), Flax (*Linum usitatissimum*), Milk Thistle (*Silybum marianum*), Perilla (*Perilla frutescens*), Borage (*Borago officinalis*) and Black Cumin (*Nigella sativa*). About half of the oils were produced under our supervision, the others were purchased on the market.

Analytical method. Sample preparation was performed in accordance with a published protocol[a,b]. As regards the instrumentation it was used the dual detector gas chromatography technique: GC-FID (Gas Chromatography - Flame Ionization Detection) and GC-MS (Gas Chromatography - Mass Spectrometry). Results were validated by analyzing the NIST Reference Material *8183-3 Flax seed oil*.

[a]*Lipids* 47 (2012) 741-753
[b] *Molecules* 26 (2021) article number 5015

Tables S1-S7: Detailed fatty acid composition expressed as a percentage

Tables S8-S14: Detailed fatty acid composition expressed as mg per g of oil

Figures S1-S5: Gas chromatograms and mass spectra

TABLE S1. Hemp seed oil: 'Futura' variety produced in the Marche region, Italy (%)

	analysis1	analysis2	analysis3	**mean**	**±**	**2σ**		
14:0	0.04	0.05	0.05	**0.05**	**±**	**0.01**	14:0	
15:0	0.02	0.02	0.02	**0.02**	**±**	**0.002**	15:0	
16:0	6.77	7.09	6.84	**6.90**	**±**	**0.34**	16:0	
16:1 ω-7	0.12	0.12	0.12	**0.12**	**±**	**0.01**	16:1 ω-7	
17:0	0.05	0.05	0.05	**0.05**	**±**	**0.004**	17:0	
18:0	2.12	2.08	2.10	**2.10**	**±**	**0.05**	18:0	
18:1 ω-9	11.42	11.25	11.36	**11.34**	**±**	**0.17**	18:1 ω-9	
18:1 ω-7	0.91	0.90	0.91	**0.91**	**±**	**0.01**	18:1 ω-7	
18:2 ω-6	54.69	54.53	54.70	**54.64**	**±**	**0.19**	18:2 ω-6	LA
18:3 ω-6	4.42	4.44	4.41	**4.42**	**±**	**0.03**	18:3 ω-6	
18:3 ω-3	16.47	16.50	16.44	**16.47**	**±**	**0.06**	18:3 ω-3	ALA
18:4 ω-3	1.40	1.42	1.40	**1.41**	**±**	**0.02**	18:4 ω-3	
20:0	0.62	0.59	0.62	**0.61**	**±**	**0.03**	20:0	
20:1 ω-9	0.33	0.31	0.32	**0.32**	**±**	**0.02**	20:1 ω-9	
20:2 ω-6	0.07	0.06	0.07	**0.07**	**±**	**0.01**	20:2 ω-6	
21:0	0.01	0.01	0.01	**0.01**	**±**	**0.004**	21:0	
22:0	0.22	0.21	0.23	**0.22**	**±**	**0.02**	22:0	
22:1 ω-9	0.02	0.02	0.03	**0.02**	**±**	**0.001**	22:1 ω-9	
23:0	0.02	0.02	0.02	**0.02**	**±**	**0.003**	23:0	
24:0	0.07	0.08	0.08	**0.07**	**±**	**0.02**	24:0	
others	0.23	0.24	0.23	**0.23**	**±**	**0.01**	others	
total	100.00	100.00	100.00	100.00	**±**	0.00		

LA	54.64	±	0.19	%
ALA	16.47	±	0.06	%
LA/ALA	3.32	±	0.02	
∑ ω6	59.13	±	0.19	%
∑ ω3	17.87	±	0.06	%
∑ω6/∑ω3	3.31	±	0.02	
∑ PUFA	77.00	±	0.20	%
∑ MUFA	12.71	±	0.17	%
∑ SFA	10.05	±	0.34	%

TABLE S2. Hemp seed oil: 'Codimono' variety produced in the Veneto region, Italy (%)

	analysis1	analysis2	analysis3	**mean**	**±**	**2σ**		
12:0	0.002	0.003	0.004	**0.003**	**±**	**0.002**	12:0	
14:0	0.04	0.04	0.04	**0.04**	**±**	**0.004**	14:0	
15:0	0.02	0.02	0.02	**0.02**	**±**	**0.001**	15:0	
16:0	6.24	6.57	6.40	**6.40**	**±**	**0.323**	16:0	
16:1 ω-7	0.11	0.11	0.11	**0.11**	**±**	**0.008**	16:1 ω-7	
17:0	0.04	0.04	0.04	**0.04**	**±**	**0.002**	17:0	
18:0	1.87	1.93	1.80	**1.87**	**±**	**0.123**	18:0	
18:1 ω-9	10.30	10.40	10.06	**10.25**	**±**	**0.347**	18:1 ω-9	
18:1 ω-7	0.92	0.91	0.94	**0.92**	**±**	**0.024**	18:1 ω-7	
18:2 ω-6	55.74	55.31	54.86	**55.30**	**±**	**0.880**	18:2 ω-6	LA
18:3 ω-6	1.30	1.30	2.14	**1.58**	**±**	**0.973**	18:3 ω-6	
18:3 ω-3	21.35	21.19	21.03	**21.19**	**±**	**0.320**	18:3 ω-3	ALA
18:4 ω-3	0.53	0.53	0.85	**0.64**	**±**	**0.364**	18:4 ω-3	
20:0	0.53	0.54	0.50	**0.52**	**±**	**0.037**	20:0	
20:1 ω-9	0.33	0.39	0.37	**0.36**	**±**	**0.056**	20:1 ω-9	
20:2 ω-6	0.05	0.06	0.06	**0.06**	**±**	**0.005**	20:2 ω-6	
21:0	0.01	0.03	0.01	**0.02**	**±**	**0.018**	21:0	
22:0	0.20	0.20	0.18	**0.19**	**±**	**0.019**	22:0	
22:1 ω-9	0.02	0.02	0.03	**0.03**	**±**	**0.004**	22:1 ω-9	
23:0	0.03	0.03	0.02	**0.02**	**±**	**0.006**	23:0	
24:0	0.09	0.08	0.07	**0.08**	**±**	**0.015**	24:0	
24:1 ω-9	0.01	0.01	0.01	**0.01**	**±**	**0.003**	24:1 ω-9	
others	0.27	0.31	0.47	**0.35**	**±**	**0.205**	others	
total	100.00	100.00	100.00	100.00	±	0.000		

LA	55.30	±	0.88	%
ALA	21.19	±	0.32	%
LA/ALA	2.61	±	0.08	
∑ ω6	56.94	±	1.31	%
∑ ω3	21.82	±	0.49	%
∑ω6/∑ω3	2.61	±	0.12	
∑ PUFA	78.76	±	1.40	%
∑ MUFA	11.69	±	0.35	%
∑ SFA	9.20	±	0.35	%

TABLE S3. Flax seed oil produced in the Marche region, Italy (%)

	analysis1	analysis2	analysis3	mean	±	2σ		
12:0	0.004	0.004	0.005	**0.004**	±	**0.001**	12:0	
14:0	0.03	0.03	0.04	**0.03**	±	**0.01**	14:0	
15:0	0.01	0.01	0.02	**0.01**	±	**0.003**	15:0	
16:0	5.69	5.91	5.60	**5.73**	±	**0.32**	16:0	
16:1 ω-7	0.07	0.07	0.07	**0.07**	±	**0.01**	16:1 ω7	
17:0	0.04	0.04	0.04	**0.04**	±	**0.001**	17:0	
18:0	2.91	2.94	2.95	**2.93**	±	**0.04**	18:0	
18:1 ω-9	12.71	12.92	13.03	**12.89**	±	**0.32**	18:1 ω9	
18:1 ω-7	1.38	1.40	1.65	**1.48**	±	**0.30**	18:1 ω7	
18:2 ω-6	11.55	11.55	11.56	**11.55**	±	**0.01**	18:2 ω6	LA
19:0	0.01	0.02	0.01	**0.02**	±	**0.01**	19:0	
18:3 ω-3	64.01	63.45	63.23	**63.56**	±	**0.80**	18:3 ω3	ALA
18:4 ω-3	0.04	0.04	0.03	**0.04**	±	**0.01**	18:4 ω3	
20:0	0.07	0.08	0.08	**0.08**	±	**0.02**	20:0	
20:1 ω-11	0.01	0.01	0.01	**0.01**	±	**0.001**	20:1 ω11	
20:1 ω-9	0.24	0.27	0.26	**0.26**	±	**0.03**	20:1 ω9	
20:2 ω-6	0.03	0.06	0.05	**0.05**	±	**0.03**	20:2 ω-6	
21:0	0.002	0.002	0.001	**0.001**	±	**0.001**	21:0	
22:0	0.08	0.09	0.10	**0.09**	±	**0.01**	22:0	
22:1 ω-9	0.71	0.73	0.75	**0.73**	±	**0.04**	22:1 ω9	
23:0	0.01	0.01	0.01	**0.01**	±	**0.003**	23:0	
24:0	0.03	0.03	0.03	**0.03**	±	**0.001**	24:0	
24:1 ω-9	0.03	0.03	0.03	**0.03**	±	**0.003**	24:1 ω-9	
others	0.33	0.32	0.44	**0.36**	±	**0.14**	others	
total	100.00	100.00	100.00	100.00	±	0.000		

LA	11.55	±	0.01	%
ALA	63.56	±	0.80	%
LA/ALA	0.18	±	0.003	
∑ ω6	11.60	±	0.03	%
∑ ω3	63.60	±	0.80	%
∑ω6/∑ω3	0.18	±	0.003	
∑ PUFA	75.20	±	0.80	%
∑ MUFA	15.46	±	0.44	%
∑ SFA	8.98	±	0.32	%

TABLE S4. Milk Thistle seed oil produced in the Marche region, Italy (%)

	analysis1	analysis2	analysis3	**mean**	**±**	**2σ**		
12:0	0.005	0.004	0.01	**0.005**	**±**	**0.001**	12:0	
14:0	0.08	0.08	0.09	**0.08**	**±**	**0.011**	14:0	
15:0	0.01	0.02	0.02	**0.02**	**±**	**0.002**	15:0	
16:0	8.32	8.30	8.49	**8.37**	**±**	**0.216**	16:0	
16:1 ω-7	0.08	0.08	0.09	**0.08**	**±**	**0.009**	16:1 ω7	
17:0	0.07	0.07	0.07	**0.07**	**±**	**0.005**	17:0	
18:0	3.95	3.97	4.03	**3.98**	**±**	**0.080**	18:0	
18:1 ω-9	31.36	32.61	31.91	**31.96**	**±**	**1.248**	18:1 ω9	
18:1 ω-7	1.43	0.40	0.41	**0.75**	**±**	**1.177**	18:1 ω7	
18:2 ω-6	48.37	48.00	48.16	**48.17**	**±**	**0.373**	18:2 ω6	LA
19:0	0.03	0.02	0.02	**0.02**	**±**	**0.005**	19:0	
18:3 ω-3	0.27	0.31	0.29	**0.29**	**±**	**0.037**	18:3 ω3	ALA
20:0	2.49	2.47	2.48	**2.48**	**±**	**0.015**	20:0	
20:1 ω-9	0.94	0.91	1.20	**1.02**	**±**	**0.311**	20:1 ω9	
20:2 ω-6	0.03	0.03	0.03	**0.03**	**±**	**0.004**	20:2 ω-6	
21:0	0.02	0.02	0.02	**0.02**	**±**	**0.0004**	21:0	
22:0	1.67	1.72	1.74	**1.71**	**±**	**0.075**	22:0	
22:1 ω-9	0.16	0.17	0.17	**0.16**	**±**	**0.013**	22:1 ω9	
23:0	0.02	0.05	0.02	**0.03**	**±**	**0.032**	23:0	
24:0	0.41	0.43	0.43	**0.42**	**±**	**0.029**	24:0	
24:1 ω-9	0.01	0.06	0.02	**0.03**	**±**	**0.052**	24:1 ω-9	
others	0.29	0.28	0.30	**0.29**	**±**	**0.024**	others	
total	100.00	100.00	100.00	100.00	±	0.000		

LA	48.17	±	0.37	%
ALA	0.29	±	0.04	%
LA/ALA	167.06	±	22.52	
∑ ω6	48.21	±	0.37	%
∑ ω3	0.29	±	0.04	%
∑ω6/∑ω3	167.18	±	22.538	
∑ PUFA	48.50	±	0.38	%
∑ MUFA	34.00	±	1.74	%
∑ SFA	17.21	±	0.25	%

TABLE S5. Perilla seed oil purchased on the market (%)

	analysis1	analysis2	analysis3	mean	±	2σ		
12:0	0.003	0.004	0.004	**0.004**	±	**0.001**	12:0	
14:0	0.03	0.03	0.03	**0.03**	±	**0.002**	14:0	
15:0	0.01	0.01	0.01	**0.01**	±	**0.001**	15:0	
16:0	5.38	5.21	5.35	**5.31**	±	**0.180**	16:0	
16:1 ω-7	0.06	0.06	0.06	**0.06**	±	**0.002**	16:1 n7	
17:0	0.03	0.03	0.03	**0.03**	±	**0.002**	17:0	
18:0	2.18	2.17	2.19	**2.18**	±	**0.014**	18:0	
18:1 ω-9	14.90	13.67	14.51	**14.36**	±	**1.258**	18:1 n9	
18:1 ω-7	0.66	1.17	0.65	**0.83**	±	**0.595**	18:1 n7	
18:2 ω-6	13.46	13.59	13.62	**13.56**	±	**0.175**	18:2 n6	LA
19:0	0.01	0.01	0.01	**0.01**	±	**0.005**	19:0	
18:3 ω-3	62.30	62.91	62.68	**62.63**	±	**0.609**	18:3 n3	ALA
18:4 ω-3	0.04	0.03	0.03	**0.03**	±	**0.016**	18:4 n3	
20:0	0.09	0.09	0.09	**0.09**	±	**0.003**	20:0	
20:1 ω-9	0.09	0.09	0.10	**0.09**	±	**0.011**	20:1 n9	
20:2 ω-6	0.03	0.17	0.13	**0.11**	±	**0.147**	20:2 n6	
21:0	0.004	0.002	0.003	**0.003**	±	**0.002**	21:0	
22:0	0.05	0.05	0.05	**0.05**	±	**0.007**	22:0	
23:0	0.01	0.01	0.01	**0.01**	±	**0.004**	23:0	
24:0	0.03	0.04	0.03	**0.03**	±	**0.016**	24:0	
others	0.62	0.65	0.42	**0.56**	±	**0.246**	others	
total	100.00	100.00	100.00	100.00	±	0.000		

LA	13.56	±	0.18	%
ALA	62.63	±	0.61	%
LA/ALA	0.22	±	0.005	
∑ ω6	13.66	±	0.23	%
∑ ω3	62.66	±	0.61	%
∑ω6/∑ω3	0.22	±	0.006	
∑ PUFA	76.33	±	0.65	%
∑ MUFA	15.34	±	1.39	%
∑ SFA	7.77	±	0.18	%

TABLE S6. Borage seed oil purchased on the market (%)

	analysis1	analysis2	analysis3	mean	±	2σ		
12:0	0.003	0.003	0.004	**0.003**	±	**0.001**	12:0	
14:0	0.06	0.07	0.07	**0.07**	±	**0.01**	14:0	
15:0	0.01	0.01	0.01	**0.01**	±	**0.001**	15:0	
16:0	10.37	10.55	10.67	**10.53**	±	**0.29**	16:0	
16:1 ω-7	0.14	0.15	0.16	**0.15**	±	**0.02**	16:1 n7	
17:0	0.04	0.04	0.04	**0.04**	±	**0.002**	17:0	
18:0	3.37	3.25	3.17	**3.26**	±	**0.20**	18:0	
18:1 ω-9	16.10	16.07	15.90	**16.02**	±	**0.21**	18:1 n9	
18:1 ω-7	0.87	0.71	0.67	**0.75**	±	**0.21**	18:1 n7	
18:2 ω-6	38.08	38.50	38.64	**38.41**	±	**0.59**	18:2 n6	LA
18:3 ω-6	22.54	23.07	23.35	**22.98**	±	**0.82**	18:3 n6	
18:3 ω-3	0.26	0.22	0.22	**0.23**	±	**0.05**	18:3 n3	ALA
18:4 ω-3	0.13	0.15	0.14	**0.14**	±	**0.02**	18:4 n3	
20:0	0.23	0.21	0.29	**0.24**	±	**0.08**	20:0	
20:1 ω-9	3.71	3.45	3.32	**3.50**	±	**0.40**	20:1 n9	
20:2 ω-6	0.16	0.15	0.15	**0.15**	±	**0.01**	20:2 n6	
22:0	0.12	0.10	0.10	**0.11**	±	**0.03**	22:0	
22:1 ω-9	2.34	1.98	1.87	**2.07**	±	**0.49**	22:1 ω9	
23:0	0.01	0.01	0.01	**0.01**	±	**0.004**	23:0	
24:0	0.04	0.05	0.04	**0.04**	±	**0.01**	24:0	
24:1 ω-9	1.17	1.00	0.93	**1.03**	±	**0.25**	24:1 ω-9	
others	0.24	0.26	0.24	**0.24**	±	**0.02**	others	
total	100.00	100.00	100.00	100.00	±	0.00		

LA	38.41	±	0.59	%
ALA	0.23	±	0.05	%
LA/ALA	164.76	±	35.74	
∑ ω6	61.54	±	1.01	%
∑ ω3	0.37	±	0.05	%
∑ω6/∑ω3	165.26	±	25.20	
∑ PUFA	61.91	±	1.01	%
∑ MUFA	23.53	±	0.74	%
∑ SFA	14.32	±	0.37	%

TABLE S7. Black Cumin seed oil purchased on the market (%)

	analysis1	analysis2	analysis3	mean	±	2σ		
12:0	0.01	0.01	0.005	**0.005**	±	**0.001**	12:0	
14:0	0.15	0.15	0.15	**0.15**	±	**0.002**	14:0	
15:0	0.02	0.03	0.03	**0.03**	±	**0.002**	15:0	
16:0	11.23	11.29	11.25	**11.26**	±	**0.06**	16:0	
16:1 ω-7	0.17	0.17	0.17	**0.17**	±	**0.01**	16:1 n7	
17:0	0.06	0.06	0.05	**0.06**	±	**0.002**	17:0	
18:0	2.68	2.62	2.58	**2.63**	±	**0.10**	18:0	
18:1 ω-9	21.19	20.94	21.07	**21.06**	±	**0.25**	18:1 n9	
18:1 ω-7	1.29	1.40	1.38	**1.36**	±	**0.12**	18:1 n7	
18:2 ω-6	59.05	59.37	59.42	**59.28**	±	**0.40**	18:2 n6	LA
19:0	0.04	0.03	0.03	**0.03**	±	**0.01**	19:0	
18:3 ω-3	0.25	0.25	0.28	**0.26**	±	**0.03**	18:3 n3	ALA
20:0	0.18	0.18	0.17	**0.18**	±	**0.01**	20:0	
20:1 ω-9	0.39	0.38	0.37	**0.38**	±	**0.02**	20:1 n9	
20:2 ω-6	2.87	2.76	2.70	**2.78**	±	**0.17**	20:2 n6	
22:0	0.03	0.03	0.03	**0.03**	±	**0.002**	22:0	
22:1 ω-9	0.08	0.07	0.07	**0.07**	±	**0.01**	22:1 ω9	
23:0	0.01	0.01	0.01	**0.01**	±	**0.0003**	23:0	
24:0	0.003	0.005	0.002	**0.003**	±	**0.003**	24:0	
others	0.33	0.27	0.24	**0.28**	±	**0.09**	others	
total	100.00	100.00	100.00	100.00	±	0.00		

LA	59.28	±	0.40	%
ALA	0.26	±	0.03	%
LA/ALA	228.78	±	31.65	
∑ ω6	62.06	±	0.44	%
∑ ω3	0.26	±	0.03	%
∑ω6/∑ω3	239.50	±	33.19	
∑ PUFA	62.32	±	0.44	%
∑ MUFA	23.04	±	0.28	%
∑ SFA	14.37	±	0.12	%

TABLE S8. Hemp seed oil: 'Futura' variety produced in the Marche region, Italy (mg/g)

	analysis1	analysis2	analysis3	**mean**	**±**	**2σ**		
14:0	0.41	0.49	0.46	**0.45**	**±**	**0.08**	14:0	
15:0	0.17	0.20	0.19	**0.19**	**±**	**0.02**	15:0	
16:0	63.58	66.93	64.52	**65.01**	**±**	**3.46**	16:0	
16:1 ω-7	1.08	1.16	1.18	**1.14**	**±**	**0.10**	16:1 ω-7	
17:0	0.45	0.46	0.43	**0.45**	**±**	**0.03**	17:0	
18:0	19.94	19.60	19.81	**19.78**	**±**	**0.34**	18:0	
18:1 ω-9	107.20	106.21	107.19	**106.87**	**±**	**1.14**	18:1 ω-9	
18:1 ω-7	8.52	8.52	8.56	**8.53**	**±**	**0.05**	18:1 ω-7	
18:2 ω-6	513.55	514.79	516.36	**514.90**	**±**	**2.81**	18:2 ω-6	LA
18:3 ω-6	41.54	41.87	41.59	**41.67**	**±**	**0.36**	18:3 ω-6	
18:3 ω-3	154.62	155.75	155.17	**155.18**	**±**	**1.13**	18:3 ω-3	ALA
18:4 ω-3	13.11	13.38	13.25	**13.25**	**±**	**0.27**	18:4 ω-3	
20:0	5.82	5.61	5.82	**5.75**	**±**	**0.25**	20:0	
20:1 ω-9	3.05	2.91	3.06	**3.01**	**±**	**0.17**	20:1 ω-9	
20:2 ω-6	0.62	0.58	0.68	**0.63**	**±**	**0.11**	20:2 ω-6	
21:0	0.10	0.12	0.14	**0.12**	**±**	**0.04**	21:0	
22:0	2.03	2.00	2.15	**2.06**	**±**	**0.16**	22:0	
22:1 ω-9	0.23	0.22	0.24	**0.23**	**±**	**0.01**	22:1 ω-9	
23:0	0.20	0.22	0.23	**0.22**	**±**	**0.03**	23:0	
24:0	0.61	0.73	0.76	**0.70**	**±**	**0.16**	24:0	
others	2.16	2.25	2.20	**2.20**	**±**	**0.09**	others	
total fatty acids	939,00	944,00	944,00	942,33	±	5,77		

LA	514.90	±	2.81	mg/g
ALA	155.18	±	1.13	mg/g
LA/ALA	3.32	±	0.04	
∑ ω6	557.20	±	2.84	mg/g
∑ ω3	168.43	±	1.16	mg/g
∑ω6/∑ω3	3.31	±	0.04	
∑ PUFA	725.63	±	3.07	mg/g
∑ MUFA	119.78	±	1.16	mg/g
∑ SFA	94.72	±	3.49	mg/g

TABLE S9. Hemp seed oil: 'Codimono' variety produced in the Veneto region, Italy (mg/g)

	analysis1	analysis2	analysis3	**mean**	**±**	**2σ**		
12:0	0.02	0.02	0.04	**0.03**	**±**	**0.02**	12:0	
14:0	0.36	0.40	0.39	**0.38**	**±**	**0.04**	14:0	
15:0	0.15	0.16	0.16	**0.15**	**±**	**0.01**	15:0	
16:0	57.31	60.73	59.46	**59.17**	**±**	**3.46**	16:0	
16:1 ω-7	1.01	1.06	0.99	**1.02**	**±**	**0.07**	16:1 ω-7	
17:0	0.33	0.34	0.35	**0.34**	**±**	**0.02**	17:0	
18:0	17.15	17.82	16.75	**17.24**	**±**	**1.08**	18:0	
18:1 ω-9	94.56	96.18	93.46	**94.73**	**±**	**2.73**	18:1 ω-9	
18:1 ω-7	8.47	8.45	8.70	**8.54**	**±**	**0.28**	18:1 ω-7	
18:2 ω-6	511.70	511.63	509.66	**511.00**	**±**	**2.31**	18:2 ω-6	LA
18:3 ω-6	11.94	12.01	19.90	**14.61**	**±**	**9.15**	18:3 ω-6	
18:3 ω-3	196.00	195.96	195.37	**195.78**	**±**	**0.71**	18:3 ω-3	ALA
18:4 ω-3	4.88	4.89	7.86	**5.87**	**±**	**3.43**	18:4 ω-3	
20:0	4.84	4.97	4.66	**4.82**	**±**	**0.31**	20:0	
20:1 ω-9	3.05	3.59	3.40	**3.35**	**±**	**0.55**	20:1 ω-9	
20:2 ω-6	0.49	0.54	0.53	**0.52**	**±**	**0.05**	20:2 ω-6	
21:0	0.11	0.26	0.13	**0.16**	**±**	**0.17**	21:0	
22:0	1.80	1.84	1.69	**1.78**	**±**	**0.16**	22:0	
22:1 ω-9	0.22	0.23	0.26	**0.24**	**±**	**0.04**	22:1 ω-9	
23:0	0.23	0.26	0.20	**0.23**	**±**	**0.06**	23:0	
24:0	0.78	0.72	0.65	**0.72**	**±**	**0.13**	24:0	
24:1 ω-9	0.09	0.10	0.08	**0.09**	**±**	**0.03**	24:1 ω-9	
others	2.51	2.84	4.32	**3.22**	**±**	**1.93**	others	
total fatty acids	918.00	925.00	929.00	924.00	±	11.14		

LA	511.00	±	2.31	mg/g
ALA	195.78	±	0.71	mg/g
LA/ALA	2.61	±	0.02	
∑ ω6	526.13	±	9.44	mg/g
∑ ω3	201.65	±	3.50	mg/g
∑ω6/∑ω3	2.61	±	0.09	
∑ PUFA	727.78	±	10.07	mg/g
∑ MUFA	107.97	±	2.80	mg/g
∑ SFA	85.02	±	3.65	mg/g

TABLE S10. Flax seed oil produced in the Marche region, Italy (mg/g)

	analysis1	analysis2	analysis3	mean	±	2σ		
12:0	0.03	0.04	0.04	**0.04**	±	**0.01**	12:0	
14:0	0.29	0.32	0.35	**0.32**	±	**0.06**	14:0	
15:0	0.12	0.13	0.14	**0.13**	±	**0.02**	15:0	
16:0	53.14	55.25	52.44	**53.61**	±	**2.92**	16:0	
16:1 ω-7	0.62	0.67	0.70	**0.66**	±	**0.08**	16:1 ω7	
17:0	0.34	0.36	0.36	**0.35**	±	**0.01**	17:0	
18:0	27.22	27.48	27.63	**27.44**	±	**0.42**	18:0	
18:1 ω-9	118.73	120.77	121.93	**120.48**	±	**3.23**	18:1 ω9	
18:1 ω-7	12.92	13.09	15.45	**13.82**	±	**2.83**	18:1 ω7	
18:2 ω-6	107.86	107.96	108.20	**108.00**	±	**0.35**	18:2 ω6	LA
19:0	0.11	0.18	0.13	**0.14**	±	**0.07**	19:0	
18:3 ω-3	597.83	593.25	591.86	**594.31**	±	**6.25**	18:3 ω3	ALA
18:4 ω-3	0.40	0.40	0.32	**0.37**	±	**0.10**	18:4 ω3	
20:0	0.62	0.71	0.78	**0.71**	±	**0.16**	20:0	
20:1 ω-11	0.07	0.08	0.07	**0.07**	±	**0.01**	20:1 ω11	
20:1 ω-9	2.23	2.49	2.48	**2.40**	±	**0.30**	20:1 ω9	
20:2 ω-6	0.29	0.54	0.45	**0.42**	±	**0.26**	20:2 ω-6	
21:0	0.01	0.02	0.01	**0.01**	±	**0.01**	21:0	
22:0	0.77	0.83	0.89	**0.83**	±	**0.12**	22:0	
22:1 ω-9	6.63	6.85	7.00	**6.83**	±	**0.38**	22:1 ω9	
23:0	0.11	0.08	0.08	**0.09**	±	**0.03**	23:0	
24:0	0.31	0.32	0.33	**0.32**	±	**0.01**	24:0	
24:1 ω-9	0.26	0.24	0.24	**0.25**	±	**0.02**	24:1 ω-9	
others	3.09	2.97	4.14	**3.40**	±	**1.28**	others	
total fatty acids	934.00	935.00	936.00	935.00	±	2.00		

LA	108.00	±	0.35	mg/g
ALA	594.31	±	6.25	mg/g
LA/ALA	0.18	±	0.002	
∑ ω6	108.43	±	0.43	mg/g
∑ ω3	594.68	±	6.25	mg/g
∑ω6/∑ω3	0.18	±	0.003	
∑ PUFA	703.11	±	6.27	mg/g
∑ MUFA	144.50	±	4.32	mg/g
∑ SFA	83.99	±	2.96	mg/g

TABLE S11. Milk Thistle seed oil produced in the Marche region, Italy (mg/g)

	analysis1	analysis2	analysis3	mean	±	2σ		
12:0	0.05	0.04	0.05	**0.05**	**±**	**0.01**	12:0	
14:0	0.74	0.76	0.83	**0.78**	**±**	**0.10**	14:0	
15:0	0.14	0.14	0.16	**0.15**	**±**	**0.02**	15:0	
16:0	79.08	78.89	80.75	**79.57**	**±**	**2.05**	16:0	
16:1 ω-7	0.74	0.78	0.82	**0.78**	**±**	**0.08**	16:1 ω7	
17:0	0.65	0.69	0.70	**0.68**	**±**	**0.05**	17:0	
18:0	37.54	37.80	38.28	**37.87**	**±**	**0.76**	18:0	
18:1 ω-9	298.26	310.10	303.49	**303.95**	**±**	**11.87**	18:1 ω9	
18:1 ω-7	13.56	3.84	3.90	**7.10**	**±**	**11.20**	18:1 ω7	
18:2 ω-6	459.99	456.46	457.96	**458.14**	**±**	**3.55**	18:2 ω6	LA
19:0	0.25	0.20	0.22	**0.22**	**±**	**0.05**	19:0	
18:3 ω-3	2.56	2.91	2.76	**2.74**	**±**	**0.35**	18:3 ω3	ALA
20:0	23.65	23.51	23.61	**23.59**	**±**	**0.15**	20:0	
20:1 ω-9	8.93	8.70	11.37	**9.67**	**±**	**2.96**	20:1 ω9	
20:2 ω-6	0.29	0.32	0.33	**0.31**	**±**	**0.04**	20:2 ω-6	
21:0	0.19	0.20	0.20	**0.20**	**±**	**0.004**	21:0	
22:0	15.87	16.35	16.57	**16.26**	**±**	**0.71**	22:0	
22:1 ω-9	1.49	1.57	1.61	**1.56**	**±**	**0.12**	22:1 ω9	
23:0	0.22	0.49	0.22	**0.31**	**±**	**0.31**	23:0	
24:0	3.85	4.05	4.12	**4.01**	**±**	**0.28**	24:0	
24:1 ω-9	0.14	0.60	0.20	**0.31**	**±**	**0.50**	24:1 ω-9	
others	2.79	2.62	2.83	**2.75**	**±**	**0.23**	others	
total fatty acids	951.00	951.00	951.00					

LA	458.14	±	3.55	mg/g
ALA	2.74	±	0.35	mg/g
LA/ALA	167.06	±	22.52	
∑ ω6	458.45	±	3.55	mg/g
∑ ω3	2.74	±	0.35	mg/g
∑ω6/∑ω3	167.18	±	22.538	
∑ PUFA	461.19	±	3.57	mg/g
∑ MUFA	323.37	±	16.59	mg/g
∑ SFA	163.69	±	2.35	mg/g

TABLE S12. Perilla seed oil purchased on the market (mg/g)

	analysis1	analysis2	analysis3	mean	±	2σ		
12:0	0.03	0.04	0.04	**0.04**	±	**0.01**	12:0	
14:0	0.25	0.26	0.27	**0.26**	±	**0.02**	14:0	
15:0	0.10	0.09	0.09	**0.09**	±	**0.01**	15:0	
16:0	50.34	48.76	50.05	**49.72**	±	**1.69**	16:0	
16:1 ω-7	0.60	0.58	0.59	**0.59**	±	**0.01**	16:1 n7	
17:0	0.32	0.31	0.31	**0.31**	±	**0.02**	17:0	
18:0	20.42	20.34	20.48	**20.41**	±	**0.13**	18:0	
18:1 ω-9	139.47	127.96	135.84	**134.42**	±	**11.77**	18:1 n9	
18:1 ω-7	6.17	10.94	6.07	**7.73**	±	**5.57**	18:1 n7	
18:2 ω-6	125.94	127.24	127.45	**126.88**	±	**1.64**	18:2 n6	LA
19:0	0.14	0.12	0.09	**0.12**	±	**0.05**	19:0	
18:3 ω-3	583.15	588.80	586.65	**586.20**	±	**5.70**	18:3 n3	ALA
18:4 ω-3	0.39	0.26	0.25	**0.30**	±	**0.15**	18:4 n3	
20:0	0.85	0.84	0.82	**0.83**	±	**0.03**	20:0	
20:1 ω-9	0.85	0.86	0.94	**0.88**	±	**0.11**	20:1 n9	
20:2 ω-6	0.26	1.59	1.21	**1.02**	±	**1.37**	20:2 n6	
21:0	0.04	0.02	0.03	**0.03**	±	**0.02**	21:0	
22:0	0.50	0.44	0.47	**0.47**	±	**0.06**	22:0	
23:0	0.10	0.13	0.12	**0.12**	±	**0.03**	23:0	
24:0	0.25	0.38	0.25	**0.29**	±	**0.15**	24:0	
others	5.85	6.05	3.96	**5.29**	±	**2.30**	others	
total fatty acids	936.00	936.00	936.00					

LA	126.88	±	1.64	mg/g
ALA	586.20	±	5.70	mg/g
LA/ALA	0.22	±	0.005	
∑ ω6	127.90	±	2.14	mg/g
∑ ω3	586.50	±	5.70	mg/g
∑ω6/∑ω3	0.22	±	0.006	
∑ PUFA	714.40	±	6.09	mg/g
∑ MUFA	143.62	±	13.03	mg/g
∑ SFA	72.69	±	1.70	mg/g

TABLE S13. Borage seed oil purchased on the market (mg/g)

	analysis1	analysis2	analysis3	**mean**	**±**	**2σ**		
12:0	0.03	0.03	0.04	**0.03**	**±**	**0.01**	12:0	
14:0	0.60	0.67	0.69	**0.65**	**±**	**0.10**	14:0	
15:0	0.06	0.07	0.07	**0.07**	**±**	**0.01**	15:0	
16:0	97.73	99.41	100.48	**99.21**	**±**	**2.77**	16:0	
16:1 ω-7	1.34	1.45	1.49	**1.43**	**±**	**0.16**	16:1 n7	
17:0	0.40	0.38	0.38	**0.39**	**±**	**0.02**	17:0	
18:0	31.74	30.57	29.87	**30.73**	**±**	**1.89**	18:0	
18:1 ω-9	151.68	151.37	149.80	**150.95**	**±**	**2.02**	18:1 n9	
18:1 ω-7	8.24	6.70	6.36	**7.10**	**±**	**2.01**	18:1 n7	
18:2 ω-6	358.67	362.68	363.99	**361.78**	**±**	**5.55**	18:2 n6	LA
18:3 ω-6	212.30	217.30	219.94	**216.51**	**±**	**7.76**	18:3 n6	
18:3 ω-3	2.45	2.07	2.07	**2.20**	**±**	**0.44**	18:3 n3	ALA
18:4 ω-3	1.21	1.37	1.36	**1.31**	**±**	**0.18**	18:4 n3	
20:0	2.19	1.97	2.74	**2.30**	**±**	**0.79**	20:0	
20:1 ω-9	34.96	32.54	31.31	**32.94**	**±**	**3.72**	20:1 n9	
20:2 ω-6	1.48	1.43	1.42	**1.44**	**±**	**0.07**	20:2 n6	
22:0	1.16	0.98	0.90	**1.02**	**±**	**0.27**	22:0	
22:1 ω-9	22.06	18.69	17.64	**19.46**	**±**	**4.62**	22:1 ω9	
23:0	0.05	0.05	0.08	**0.06**	**±**	**0.04**	23:0	
24:0	0.38	0.47	0.38	**0.41**	**±**	**0.10**	24:0	
24:1 ω-9	11.03	9.39	8.77	**9.73**	**±**	**2.34**	24:1 ω-9	
others	2.23	2.41	2.24	**2.29**	**±**	**0.20**	others	
total fatty acids	942.00	942.00	942.00					

LA	361.78	±	5.55	mg/g
ALA	2.20	±	0.44	mg/g
LA/ALA	164.76	±	35.74	
∑ ω6	579.73	±	9.54	mg/g
∑ ω3	3.51	±	0.48	mg/g
∑ω6/∑ω3	165.26	±	25.20	
∑ PUFA	583.24	±	9.55	mg/g
∑ MUFA	221.61	±	6.99	mg/g
∑ SFA	134.86	±	3.46	mg/g

TABLE S14. Black Cumin seed oil purchased on the market (mg/g)

	analysis1	analysis2	analysis3	mean	±	2σ		
12:0	0.05	0.05	0.04	**0.04**	±	**0.01**	12:0	
14:0	1.34	1.34	1.36	**1.35**	±	**0.02**	14:0	
15:0	0.22	0.23	0.23	**0.23**	±	**0.01**	15:0	
16:0	100.93	101.49	101.15	**101.19**	±	**0.56**	16:0	
16:1 ω-7	1.51	1.54	1.57	**1.54**	±	**0.06**	16:1 n7	
17:0	0.50	0.50	0.49	**0.50**	±	**0.02**	17:0	
18:0	24.08	23.59	23.20	**23.62**	±	**0.88**	18:0	
18:1 ω-9	190.47	188.21	189.39	**189.36**	±	**2.26**	18:1 n9	
18:1 ω-7	11.58	12.60	12.37	**12.18**	±	**1.06**	18:1 n7	
18:2 ω-6	530.85	533.74	534.19	**532.93**	±	**3.62**	18:2 n6	LA
19:0	0.34	0.27	0.30	**0.31**	±	**0.07**	19:0	
18:3 ω-3	2.26	2.22	2.50	**2.33**	±	**0.31**	18:3 n3	ALA
20:0	1.62	1.58	1.54	**1.58**	±	**0.08**	20:0	
20:1 ω-9	3.48	3.38	3.32	**3.39**	±	**0.16**	20:1 n9	
20:2 ω-6	25.76	24.86	24.27	**24.96**	±	**1.51**	20:2 n6	
22:0	0.27	0.26	0.25	**0.26**	±	**0.02**	22:0	
22:1 ω-9	0.69	0.65	0.62	**0.66**	±	**0.07**	22:1 ω9	
23:0	0.06	0.06	0.06	**0.06**	±	**0.003**	23:0	
24:0	0.02	0.04	0.02	**0.03**	±	**0.02**	24:0	
others	2.94	2.39	2.15	**2.49**	±	**0.82**	others	
total fatty acids	899.00	899.00	899.00					

LA	532.93	±	3.62	mg/g
ALA	2.33	±	0.31	mg/g
LA/ALA	228.78	±	31.65	
∑ ω6	557.89	±	3.92	mg/g
∑ ω3	2.33	±	0.31	mg/g
∑ω6/∑ω3	239.50	±	33.19	
∑ PUFA	560.22	±	3.94	mg/g
∑ MUFA	207.13	±	2.50	mg/g
∑ SFA	129.16	±	1.05	mg/g

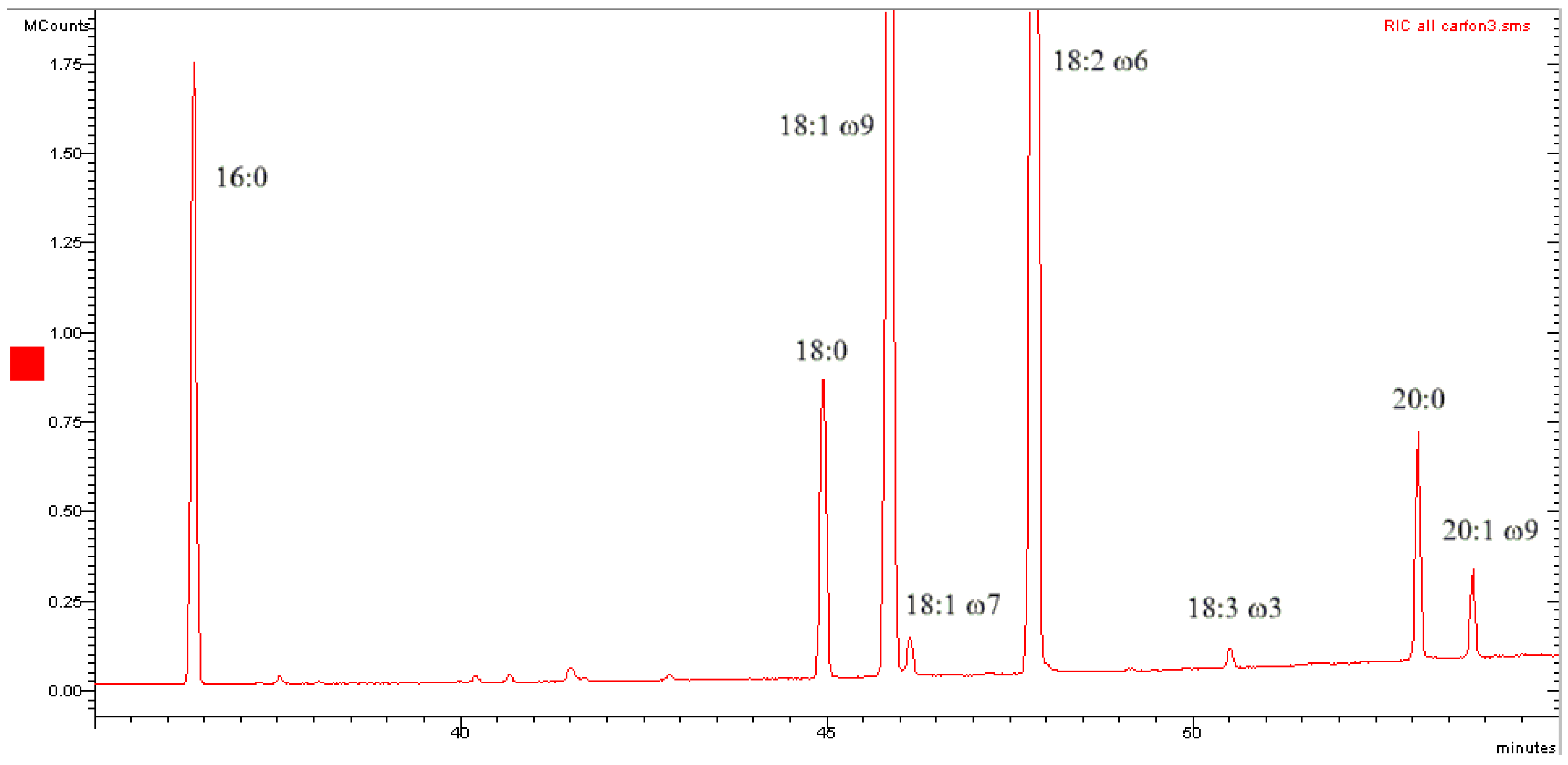

Figure S1. Gas chromatogram of the sample Milk Thistle seed oil (analysis number 3) in the time range 35-55 min obtained by Mass Spectrometry (GC-MS). Fatty acid methyl esters.

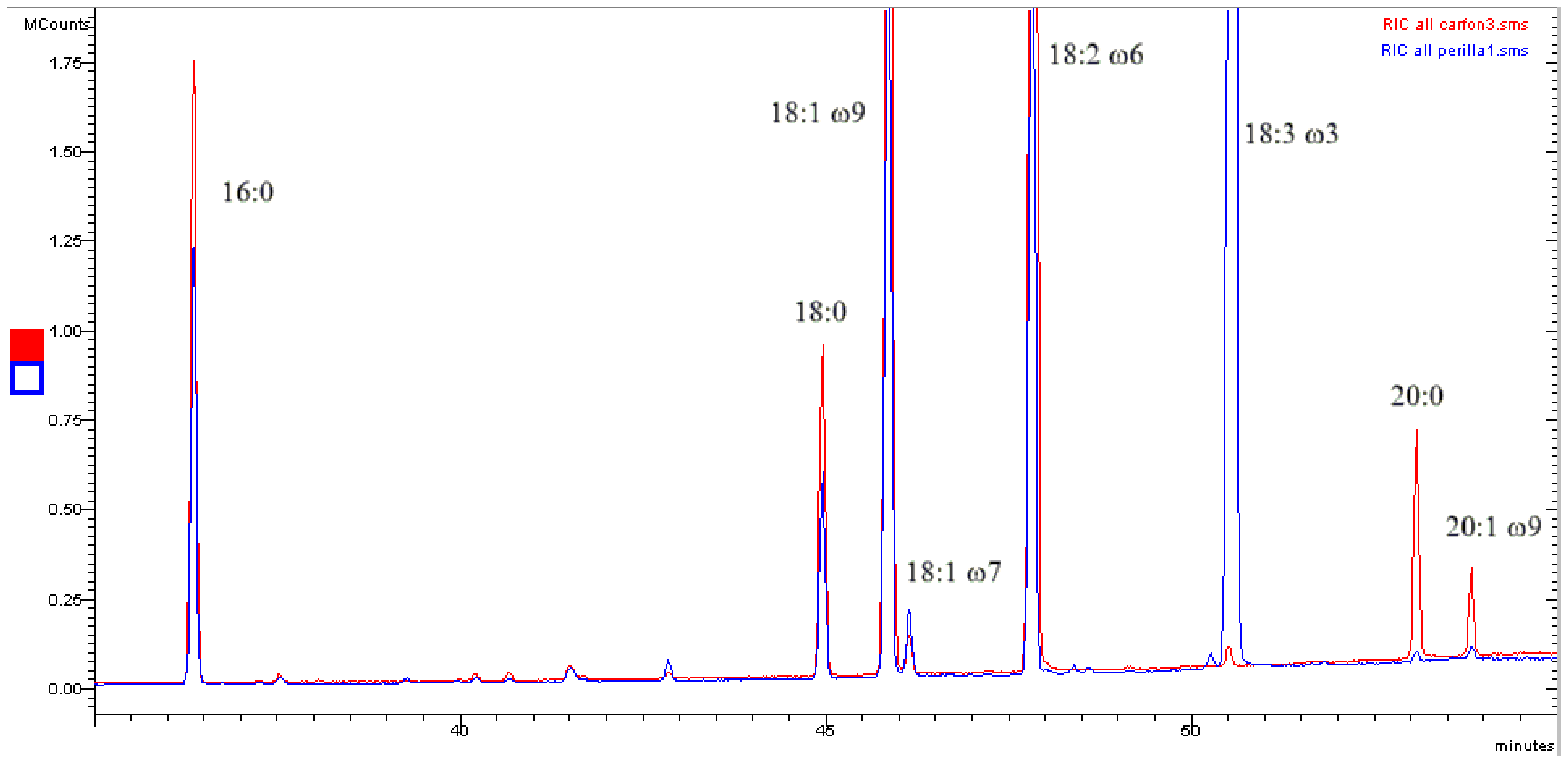

Figure S2. Gas chromatograms obtained by Mass Spectrometry (GC-MS) in the time range 35-55 min. Overlapping between the sample Milk Thistle seed oil (analysis number 3) traced in red and the sample Perilla seed oil (analysis number 1) traced in blue. Fatty acid methyl esters. Note the very different intensities of 18:3 ω3, with a high concentration in the Perilla seed oil. Differences in the concentrations of Arachidic acid (20:0) and Gondoic acid (20:1 ω9) are also observed.

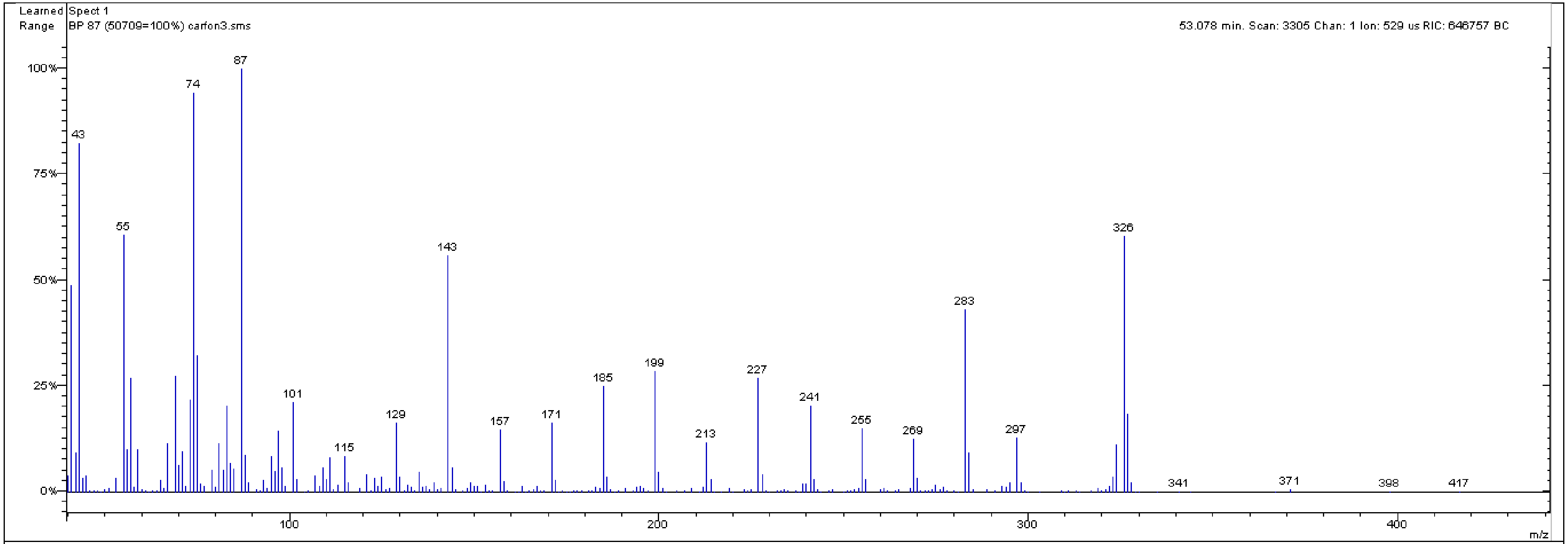

Figure S3. Mass spectrum of the peak related to 20:0 methyl ester in Milk Thistle seed oil of Figure S1. They can be seen the characteristic ionic fragments. The molecular weight of 20:0 methyl ester is 326 amu.

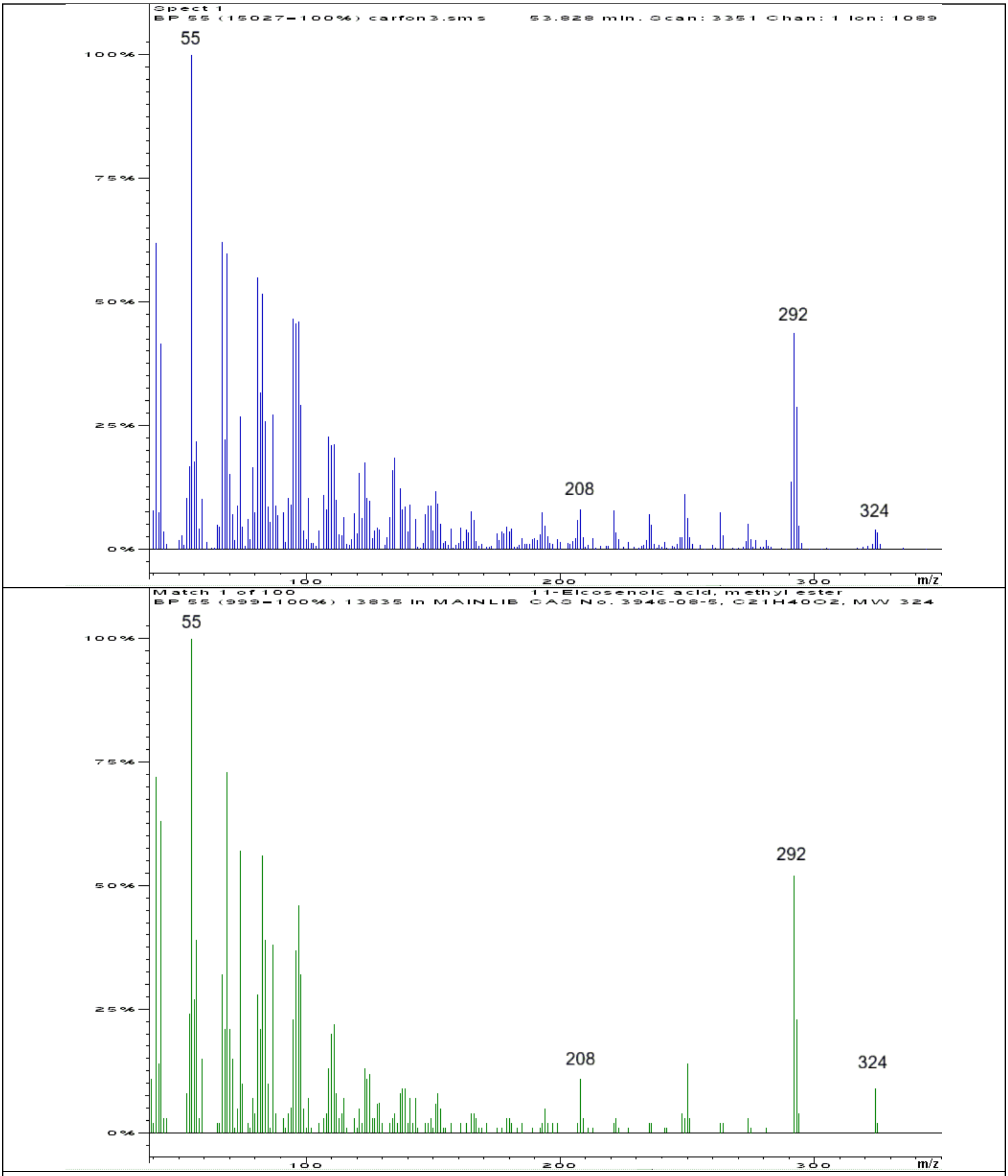

Figure S4. Top: mass spectrum of the peak related to 20:1 ω9 methyl ester in Milk Thistle seed oil of Figure S1. Bottom: mass spectrum of 20:1 ω9 methyl ester from the NIST mass spectrum database. The coincidence of the two spectra is evident. This fact, combined with the coincidence of the retention times of 20:1 ω9 methyl ester (in the sample and when injected as a pure standard) gives absolute certainty of the identification. As a further identification criterion, it should be considered that the injection of the pure standard of 20:1 ω9 methyl ester gave an identical mass spectrum to the ones showed above.

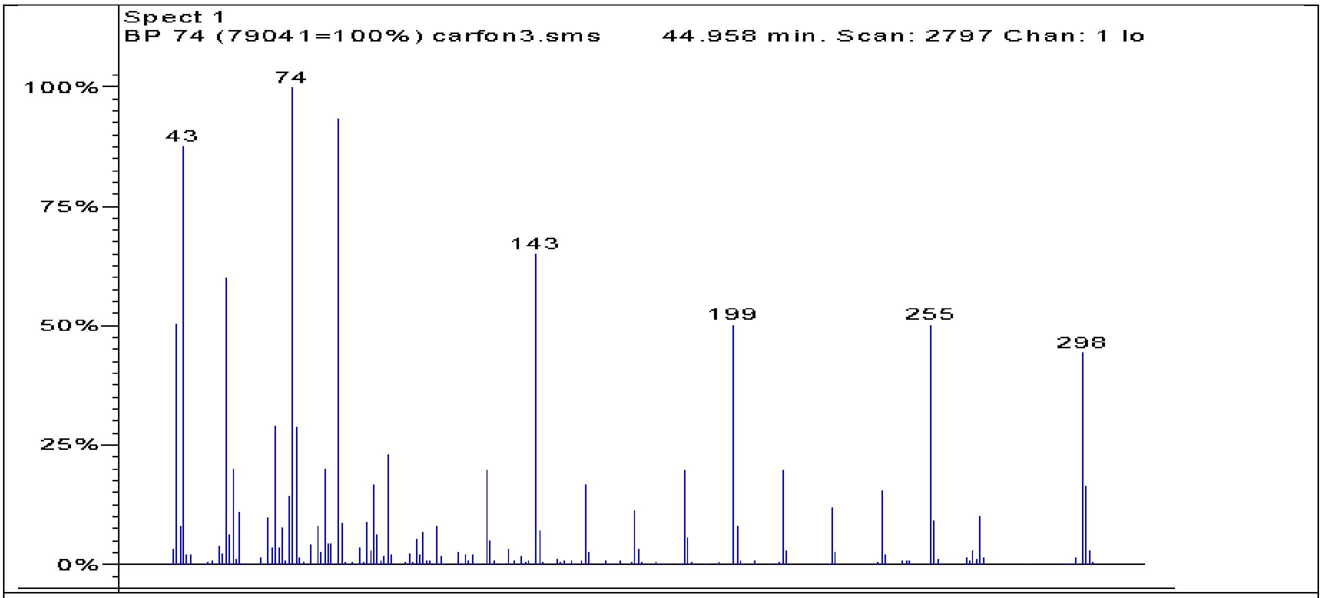

Figure S5. Mass spectrum of the peak related to 18:0 methyl ester in Milk Thistle seed oil of Figure S1.
